# Profiles of Mobile Phone Problem Use in Bullying and Cyberbullying Among Adolescents

**DOI:** 10.3389/fpsyg.2020.596961

**Published:** 2020-10-15

**Authors:** Inmaculada Méndez, Ana Belén Jorquera Hernández, Cecilia Ruiz-Esteban

**Affiliations:** Department of Evolutionary Developmental and Educational Psychology, Campus Regional Excellence Mare Nostrum, University of Murcia, Murcia, Spain

**Keywords:** bullying, cyberbullying, mobile phone, adolescence, academic adjustment

## Abstract

Technology is being used by individuals of all ages; young children show a high tendency of problematic use of devices such as smartphones. This study aimed to identify different profiles that vary in conflicts related to problematic mobile phone use and maladaptive communication and emotional patterns. Therefore, we examined whether there are significant differences in bullying and cyberbullying among teenagers who have a problem utilizing mobile phones. The study participants were 810 students of Compulsory Secondary Education (*M* = 13.99, *SD* = 1.32), with 52.2% being girls. Questionnaires on school violence and experiences related to mobile phones were administered. The latent profile analysis identified three different types of problematic mobile phone use: (a) low levels of conflict was associated with mobile phone abuse and low levels of communication and emotional use; (b) moderate levels of conflict was associated with mobile phone abuse and moderate levels of communication and emotional use; and (c) high levels of conflict was associated with mobile phone abuse and high levels of communication and emotional use. The study results highlight significant differences in the manifestations of school violence between the profiles. Finally, this study’s findings form the basis for the development of education programs to prevent mobile phone abuse and school violence and therefore academic adjustment.

## Introduction

Mobile phones have become essential in our daily lives. The mobile phone is a tool that allows communication, expression, access to information and leisure, and even aiding autonomy and sometimes prestige via generating symbolic appearances. Therefore, it fulfills various playful-expressive, referential, and communicative functions (García and Monferrer, 2009; Lee et al., 2014; Besoli et al., 2018). Additionally, social networks utilized via a mobile phone constitute a form of social interaction, allowing dynamic exchange and for users to expand their usual communication and emotional expression. Users can share feelings, opinions, and sometimes commit misconduct (doing and saying different things, including those not said in person) (Cornejo and Tapia, 2011; Fernández-Sánchez, 2013; Serrano-Puche, 2016; Díaz-López et al., 2020). Hence, mobile phone usage may damage interpersonal relationships (Lee et al., 2014).

Mobile phone usage is diverse and many aspects leading to its problematic usage must be considered (Carbonell et al., 2012a; Besoli et al., 2018). Behavioral addictions share characteristics similar to substance addictions (tolerance, comorbidity, neurobiological mechanisms, etc.). Although the DSM-5 (American Psychiatric Association [APA], 2013) proposed a category of addiction and related disorders including substance-free addictions, however, problematic mobile phone use is not yet included (Grant et al., 2010). Therefore, problematic mobile phone use should be referred to as problematic or maladaptive behaviors and not as addiction (Panova and Carbonell, 2018). Thus, the person who presents with problematic mobile phone use usually recreationally utilizes a mobile phone in excessive and uncontrollable manners. The user usually submits a short-term reward search leading to dependency and a loss of control (Cía, 2013). Hence, the person seeks to relieve their emotional discomfort (boredom, loneliness, nervousness, irritability, etc.) and utilizes the mobile phone as a buffer for emotional tension (Echeburúa and de Corral, 2010; Cía, 2013; Serrano-Puche, 2016; Santana-Vega et al., 2019). Problematic mobile phone use may sometimes lead to an over-the-over-the-face economic expense from advertising or false subscriptions. Another directly associated risk is the recording and distribution of videos and images that may be mis-used in a context of school violence, coupled with the impersonation by the intrusion of fraudulent messages, the chains of pedophiles, etc. (García and Monferrer, 2009). Mobile phone usage has been associated with unhealthy interferences in an individual’s daily life (Echeburúa and de Corral, 2010; Cía, 2013; Fernández-Sánchez, 2013; De-Sola et al., 2019). Problematic mobile phone use with the utilization of social networks, impacts negatively the physical, mental, and social well-being of the person (Lim and Yang, 2015; Amendola et al., 2019). There is evidence reported of addiction to mobile devices being greater than addiction to social media services (Barnes et al., 2019), the user profile for mobile phone addiction is different to the profile for someone addicted to the internet (De Sola-Gutiérrez et al., 2016).

Problematic mobile phone use has increased, especially during adolescence globally (Carbonell et al., 2012a; Besoli et al., 2018) and has been a subject of interest for other investigations due to their impact (Lee et al., 2014). The lack of maturity during adolescence may lead to inappropriate behaviors or attitudes even causing psychological discomfort (Besoli et al., 2018). Thus, the intensive use of mobile phones amongst adolescents has been associated with drug use, poor academic performance, low self-esteem, and poor social relations (Echeburúa and de Corral, 2010; Livingstone and Smith, 2014; Muñoz-Miralles et al., 2016); impulsivity, anxiety, and stress (De Sola-Gutiérrez et al., 2016); greater emotional mismatch (Amendola et al., 2019) and low family cohesion (Muñoz-Miralles et al., 2016; Santana-Vega et al., 2019). Teenagers above all, utilize mobile phones in recreational or communicative manners (surfing the internet, social networks, listening to music or collective fun) and rendering the mobile phone as a source of evasion, distraction, anger control or anxiety (Echeburúa and de Corral, 2010; Moral and Suárez, 2016; Díaz-López et al., 2020). Young teenagers show an inability to disconnect or turn off mobile phones that, consequently, reduces sleep, increases worry and anxiety, and is associated with a greater fear of missing out (or FOMO) on sharing experiences with others – which in turn increases the desire to use mobile phones more often (to feel connected) associated with psychological reasons leading to increases and problematic mobile use (Gil et al., 2015; Santana-Vega et al., 2019). Dependence on the social environment coupled with the need for belonging is associated with mobile phones being an essential vehicle of communication but may also become problematic and addictive (De-Sola et al., 2019). Consequently, most teenagers prefer to communicate via mobile phones rather in person because it allows them to socialize, have fun, promote their social status and identity, etc. (Moral and Suárez, 2016).

Thus, the search for identity in adolescence may mean that the media will lead to users not being aware of the risks involved in sharing information on the internet (Arab and Díaz, 2015). In adolescence there is a shared technological culture since personal information and that of personal problems, mood problems, photographs, and expectations are shared without taking the risk of the loss of privacy (Sabater Fernández and López-Hernáez, 2015). For example, “doxing” is a violation of someone’s information without their consent and is a form of cyberbullying (Chen et al., 2019). Cyberbullying occurs in a highly socialized environment. Therefore, harassment in cyberspace is linked to harassment in the face-to-face context (Casas et al., 2013; Kowalski et al., 2014; Arab and Díaz, 2015; Olweus and Limber, 2018; Chen et al., 2019). Teenagers with a problematic use of new technologies have been associated with increased bullying and cyberbullying problems (Arnaiz et al., 2016). Additionally, adolescents with problematic social media behavior are more involved in aggressive behaviors among the peers (Martínez-Ferrer et al., 2018). Previous research indicates that internet access via a mobile phone has been linked to greater involvement in the role of aggressor and in the role of victimization by cyberbullying regarding those not involved (Giménez et al., 2015; Kwok et al., 2017; Gül et al., 2019). Time spent communicating with friends, posting information, and browsing on mobile phones is associated with an increased risk of victimization by cyberbullying (Kwok et al., 2017).

Thus, our objective was to identify different profiles that vary in conflicts related to problematic mobile phone use and maladaptive communication and emotional patterns. Therefore, this study aimed to examine whether there are significant differences in bullying and cyberbullying among adolescents with problematic mobile phone behavior.

The main hypotheses are: (1) there are different profiles of problematic mobile phone use among adolescents; and (2) adolescents with mobile phone use problems will be more involved in problems of school violence (bullying and cyberbullying).

## Materials and Methods

### Participants

Initially, 1021 adolescents were recruited from secondary education centers in different geographical areas of the Region of Murcia. It is a representative sample of the secondary pupils (with a maximal error of 5%). After excluding 211 from whom informed consent was not obtained or questionnaires incomplete, 810 were finally included. Participants were from secondary education schools, with 52.2% being girls, and were 12 to 16 years old (*M* = 13.99, *SD* = 1.32). Further, 77.9% had not repeated a course and 4.8% were born outside of Spain. The distribution was homogeneous in terms of sex and age (χ2 = 4.33, *p* = 0.50) there being no differences between said sociodemographic variables. The socio-economic level of the different areas and schools was average.

#### Design and Procedure

The study protocol was approved by the Ethics Committee of the University of Murcia (ID: 2627/2019). Afterward, the participating centers from the different geographical areas of the Region of Murcia were selected. A personal interview was arranged with the management team and the educational guidance department to indicate the objectives of the study and request participation. After permission from the schools was granted, informed consent was obtained from all participants and their parents for study participation. The study instruments (detailed below) were administered during a 50-min session, maintaining anonymity and confidentiality.

### Instruments

Three assessment instruments were applied in the study. First, the following socio-demographic variables were assessed: gender (male/female), age, grade, country of birth, course repetition (yes/no), nature of the school (public/private/semi-private).

Secondly, the School Violence Questionnaire [a revision of Álvarez-García et al.’s (2011)] was administered. This included 31 items measuring the frequency of occurrence for different manifestations of school violence: violence of teachers toward students (VTS), physical indirect violence by students (VPI), physical direct violence between students (VPD), verbal violence among students (VVS), verbal violence of students toward teachers (VVT), social exclusion (SE), disruptive behavior in the classroom (DB), and violence through new information and communication technologies (VICT). The Cronbach’s α coefficient range for Álvarez-García et al.’s (2011) study was 0.67 (VPD) –0.88 (VTS). For our study, it ranged from 0.66 to 0.87, being for each factor Cronbach’s alpha: VTS (α = 0.85), VPI (α = 0.70), VPD (α = 0.72), VVS (α = 0.75), VVT (α = 0.66), SE (α = 0.73), DB (α = 0.78) and VICT (α = 0.87). Examples of items: “The students put annoying nicknames to their classmates”; “There are students who spread negative rumors about classmates and companions.”

Last, the Mobile Related Experiences Questionnaire (CERM), as prepared by Beranuy et al. (2009), was utilized. Specifically, it measures mobile phone abuse via a survey made up of 10 items on a four-point Likert scale. This instrument consists of two factors: conflicts related to mobile phone abuse (CONFLICTS) and problems due to communicational and emotional use the mobile phone (USE COMMUNICATIONAL). The Cronbach’s α coefficient 0.81 for CONFLICTS and 0.75 for USE COMMUNICATIONAL in Beranuy et al. (2009). For our study, these were, respectively, 0.91 and 0.89. Adequate reliability has been shown according to Cronbach’s alpha (α = 0.80) for the whole instrument (Beranuy et al., 2009) and for our study (α = 0.94). “Do you stop hanging out with your friend because you spend more time using your mobile?” “When you get bored, do you use the mobile as a way of distraction?”

### Data Analysis

In order to meet our objective and to be able to identify the different profiles that vary in conflicts related to problematic mobile phone use and maladaptive communication and emotional patterns, it was first necessary to perform a latent profile analysis. Specifically, in this study, latent profile analysis was utilized to identify the subgroups of students (Schreiber, 2017). After analyzing the lowest values of the Akaike Information Criterion (AIC) and the Bayesian Information Criterion (BIC), the best model was chosen (detailed below) (Muthén and Muthén, 2012). The groups of students were defined based on the different types of problematic mobile phone use: conflicts related to mobile phone abuse (CONFLICTS) and communicational and emotional use (USE COMMUNICATIONAL). To attend to the secondary objective, analysis of variance (ANOVA) was conducted to examine the different manifestations of school violence between the groups, partial eta squared (η*_*p*_^2^*) was used to estimate the magnitude of the differences and the *post hoc* test with the Bonferroni method and Cohen’s d was estimated for the magnitude of the differences (per Cohen, 1998). SSPS Statistics version 23.0 and the Excel package (XLSTAT) to run the latent class analyses was utilized.

## Results

Table 1 shows the Pearson’s correlation coefficient among the variables of this study. It demonstrates that they are all positive and statistically significant, so the profiles were analyzed.

**TABLE 1:** Pearson’s correlation coefficient between the variables of study.

**Variable**	**Conflicts**	**Use Communicational**
VTS	0.382**	0.341**
VPI	0.320**	0.294**
VPD	0.341**	0.287**
VVS	0.232**	0.270**
VVT	0.172**	0.201**
SE	0.265**	0.222**
DB	0.115**	0.169**
VICT	0.410**	0.306**

**p < 0.01.

**FIGURE 1:**
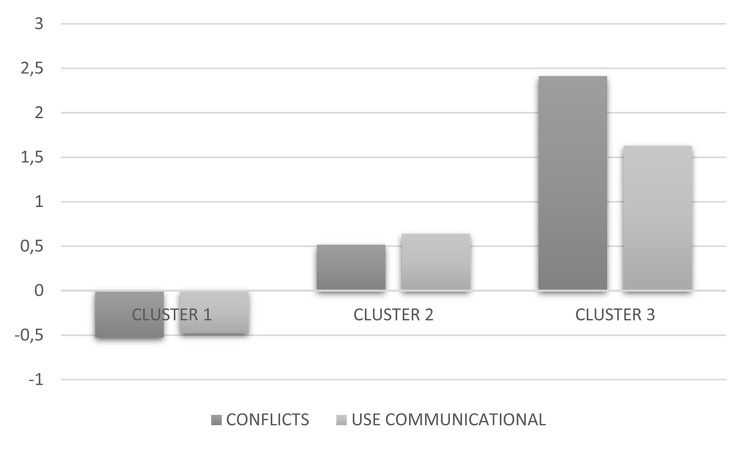
Graphical representation of the three-cluster model. Cluster 1 (non-problematic use), Cluster 2 (moderate problematic use), and Cluster 3 (problematic use).

Table 2 presents the models obtained (from two to six classes). All models were statistically significant. Model 3 present the best and the less BIC values, the best indicators of the Vuong-Lo-Mendell-Rubin likelihood-ratio test (VRT) was significant and the size was 0. The cluster and latent profile analyses identified three different types of problematic mobile phone use: (a) a first group of 534 students (65.9%), characterized by low levels of conflicts related to mobile phone abuse and low levels communicational and emotional use (termed non-problematic use); (b) a second group of 209 students (25.8%), characterized by moderate levels of conflict related to mobile phone abuse and moderate levels communicational and emotional use (termed moderate problematic use); and (c) a third group of 67 students (8.3%), characterized by high levels of conflict related to mobile phone abuse and high levels of communicational and emotional use (termed problematic use) (see Figure 1).

**TABLE 2:** The fit of the all latent class models.

**Models**	**AIC**	**BIC**	**BIC-adjusted**	**LRT**	**LRT-adjusted**	**BLRT**	**Entropy**	**Size**
2	4156.294	4189.173	4166.944	0.0000	0.0000	0.0000	0.900	0
3	4023.664	4070.635	4038.879	0.0280	0.0319	0.0000	0.781	0
4	3924.906	3985.967	3944.685	0.0000	0.0000	0.0000	0.816	1
5	3876.973	3952.125	3901.316	0.0058	0.0071	0.0000	0.870	2
6	3826.933	3916.176	3855.840	0.0000	0.0000	0.0000	0.841	2

*AIC, Akaike Information Criterion; BIC, Bayesian Information Criterion; LRT, Vuong-Lo-Mendell-Rubin likelihood-ratio test; BLRT, Bootstrap Likelihood Ratio test.*

The results of the ANOVAs revealed significant differences between the three profiles different of problematic mobile phone use regarding the manifestations of school violence (see Table 3).

**TABLE 3:** Means and standard deviations obtained by the three profiles for problematic mobile phone use and values of the partial eta squared (η*_*p*_^2^*) for each variable of school violence.

	**Group 1**		**Group 2**		**Group 3**		**Significance**		
**Variable**	***M***	***SD***	***M***	***SD***	***M***	***SD***	***F_(2,807)_***	***p***	**η *_*p*_^2^***
VTS	14.17	5.10	16.29	5.14	21.70	7.14	64.34	<0.001	0.14
VPI	6.03	2.26	7.23	2.47	8.70	3.28	46.67	<0.001	0.10
VPD	6.61	2.48	7.87	2.21	9.20	3.31	43.81	<0.001	0.10
VVS	10.97	3.33	11.88	3.20	14.08	3.72	28.25	<0.001	0.07
VVT	4.57	1.81	5.33	1.84	5.58	2.34	17.76	<0.001	0.04
SE	5.87	2.50	6.67	2.55	8.10	3.68	24.87	<0.001	0.06
DB	9.52	2.99	10.37	2.68	10.52	3.10	8.48	<0.001	0.02
VICT	8.68	3.66	10.81	4.22	15.07	7.86	74.01	<0.001	0.16

*Cluster 1 (non-problematic use), Cluster 2 (moderate problematic use), and Cluster 3 (problematic use).*

*Post hoc* comparisons (see Table 4) reported that in general, the non-problematic use group obtained significantly lower scores for the manifestations of VTS, VPI, VPD, VVS, VVT, SE, DB, and VICT than the moderate problematic use group. The former group also obtained significantly lower scores than the problematic use group. Similarly, moderate problematic use group also obtained significantly lower scores than the problematic use group, regarding the manifestations of school violence.

**TABLE 4:** Cohen’s d indexes for *post hoc* contrast groups.

**Models**	**Group1- Group 2**	**Group1- Group 3**	**Group2- Group 3**
VTS	0.41***	1.40***	0.95***
VPI	0.52***	1.12***	0.55***
VPD	0.52***	1.01***	0.56*
VVS	0.28*	0.92***	0.66***
VVT	0.42***	0.70***	−
SE	0.32*	0.84***	0.50*
DB	0.29*	0.33*	−
VICT	0.56***	1.48***	0.80***

*p < 0.05, ***p < 0.01.

## Discussion

In this study, the cluster and latent profile analyses, identified three different types of problematic mobile phone use, as described above. Similar to our study, Carbonell et al. (2012a) showed the existence of three clusters for problematic mobile phone use via the CERM, based on low, moderate or high scores for participants between 11 and 25 year old. Similarly, Díaz-López et al. (2020) showed the existence of three clusters based on the more or less adapted profile of ICT. In our study, we have shown evidence of three different conflict clusters regarding the use of mobile phones and inappropriate emotional and communication patterns, instead of taking them as one. This indicates that the objective and the first hypothesis of the study has been confirmed.

The second hypothesis of our study has been demonstrated. Our results indicate that there are significant differences in the manifestations of school violence between the profiles. Generally, in the different manifestations of school violence it has been found that the non-problematic use group showed values below the moderate problematic use group and still lower values compared to the problematic use group. The problematic use group achieved significantly higher values than the moderate problematic use group in VTS, VPI, VPP, VVS, SE, and VICT. Thus, the problematic use group is indicative of a profile of students who are at risk since they present high values of conflict with mobile use. Additionally, they indicate pattern of poor emotional adaptation and communication and hence is the most maladaptive profile compared to the other profiles obtained. The moderate problematic use group values suggest intervention to promote actions to minimize the values found and prevent the risk from increasing. Finally, the values found in the non-problematic use group are appropriate values since this is a more favorable pattern compared to the other groups. Therefore, the 67 students in the problematic use group are most at risk due to the inadequate pattern of mobile phone use and the perception of greater involvement in the different manifestations of school violence. This corroborates research showing that the problematic use of new technologies relates to greater involvement in the different manifestations of school violence (bullying and cyberbullying) (Arnaiz et al., 2016). Thus, harassment in cyberspace is linked to real-life harassment (Arab and Díaz, 2015; Olweus and Limber, 2018; Chen et al., 2019). Problematic mobile phone use behavior has been associated with an increased risk of perpetuating roles of cyber-users and cyber victims, especially from decreased awareness of shared information (Giménez et al., 2015; Kwok et al., 2017; Martínez-Ferrer et al., 2018; Gül et al., 2019). Problems in social relationships, due to problematic mobile use and poor emotional and communication adaptation, may be consequent to mobile use being connected to the teenager’s emotions and tensions (Echeburúa and de Corral, 2010; Cía, 2013; Serrano-Puche, 2016). Additionally, consequent is their search for identity (Moral and Suárez, 2016), and the preference for communication through new technologies versus that in person (Arnaiz et al., 2016) without being aware of the risks of sharing personal information on the Internet (Arab and Díaz, 2015) what can be associated with cyberbullying (Chen et al., 2019) with data that are worrying in our country.

Our study assists in educational programs to prevent problematic mobile phone use and school violence, and therefore supporting academic adjustment. Our data supports the need to promote actions aimed at improving coexistence and mobile phone use by teenagers because it may lead to problematic mobile phone use and therefore to a profile that implies a loss of control incurring problems at school and in social and family contexts (Fernández-Sánchez, 2013; Lim and Yang, 2015; Amendola et al., 2019). Hence, it would be advisable to perform actions that promote coexistence, social and communication skills, the management of emotions and stress, and healthy leisure activities (García and Monferrer, 2009; Echeburúa and de Corral, 2010; Moral and Suárez, 2016). Similarly, actions that involve family, teachers, school and orientation teams are required, creating spaces that encourage responsible mobile use (Santana-Vega et al., 2019), allow for the early detection of problematic mobile use and early risk assessment leading to school violence (García and Monferrer, 2009; Echeburúa and de Corral, 2010; Carbonell et al., 2012b; Cía, 2013; Arnaiz et al., 2016). Additionally, families monitoring and improving their relationships are also essential (Muñoz-Miralles et al., 2016; Santana-Vega et al., 2019).

Our study is limited due to it being cross-sectional and the instruments used may have been associated with the effect of social desirability. Therefore, longitudinal studies are required (Livingstone and Smith, 2014) possibly up to the university level, including, among others, gathering information on emotional management (Amendola et al., 2019), focusing on other behavioral addictions possibly associated with previous psychological problems (Echeburúa and de Corral, 2010; Cía, 2013), and to investigate family communication (Santana-Vega et al., 2019) that can provide more information to the clusters. Finally, research for the delimitation of behavioral addictions, especially in DSM-5, remains necessary to advance proper diagnosis and treatment (Grant et al., 2010; Cía, 2013).

## Data Availability Statement

The raw data supporting the conclusions of this article will be made available by the authors, without undue reservation.

## Ethics Statement

The studies involving human participants were reviewed and approved by the study protocol was approved by the Ethics Committee of the University of Murcia (ID: 2627/2019). Written informed consent to participate in this study was provided by the participants’ legal guardian/next of kin.

## Author Contributions

IM, AJ, and CR-E contributed to the conception and design of the review. IM and CR-E applied the search strategy. All authors applied the selection criteria, completed the bias-risk assessment, analyzed and the interpreted data, wrote this manuscript, and edited this manuscript.

## Conflict of Interest

The authors declare that the research was conducted in the absence of any commercial or financial relationships that could be construed as a potential conflict of interest.
